# Expensive medicines: ensuring objective appraisal and equitable access

**DOI:** 10.2471/BLT.14.148924

**Published:** 2015-01-01

**Authors:** Suzanne R Hill, Lisa Bero, Geoff McColl, Elizabeth Roughead

**Affiliations:** a University of Melbourne, Parkville, Melbourne, Victoria 3010, Australia.; b Charles Perkins Centre, University of Sydney, Sydney, Australia.; c University of South Adelaide, Adelaide, Australia.

In response to requests for the funding of new drugs, reimbursement agencies are re-evaluating some of the methods used in assessing these products. Many trials submitted for the regulatory review of new drugs do not provide adequate data for subsidy decisions. We argue that all involved in bringing medicines to market need to be explicit about the additional information required, decide how these data should be collected and assessed and the methods that should be used to set a fair price for a new drug.

In Australia, a formal appraisal of the cost–effectiveness and budget impact of a new medicine precedes any subsidy decision at national level.^1^ If a new product is subsidized, the government pays an agreed price to the manufacturer, sometimes with requirements for financial contracts to manage expenditure.^2^

Increasingly, patients are asking for early access to new drugs, particularly for treating cancer. In consequence, strategies to subsidize drugs for use under conditions of coverage with evidence development or managed entry are being proposed. A new drug might be approved even if there is no evidence to show that it satisfies the standards typically applied in health technology assessments. However, this approval is often contingent upon additional requirements for subsequent randomized trials or the collection of data on the drug’s effectiveness and safety in practice. There is no consensus on the best methods for identifying drugs appropriate for managed entry schemes, for collecting post-approval data or for the use of such data to modify decisions about coverage. 

In Australia, as in many other countries, several questions need to be answered. Can stakeholders produce a workable framework for managed entry schemes? What can be done to reduce variation in the inputs used for cost–effectiveness models? How can drug or disease registries contribute useful information to inform reimbursement decisions? How should registry data be evaluated? What can be done to make registry data more representative of the population and what types of post-progression data should be included in trials of targeted cancer therapies?

Regulatory agencies, insurers and clinicians also need to be able to determine if a new drug represents good value for money and what to do if an effective drug appears too highly priced for the benefit that it offers. The prices of several recently-introduced drugs – for example aflibercept, ivacaftor and sofosbuvir – have been questioned.^3^^-^^5^

The way in which drug prices change over time has generally been a function of the market. Typically, a new drug is launched under patent and can command a good price until the patent expires and competition and/or generic products emerge. Exceptionally, public pressure and legal challenges decreased the price of several antiretroviral drugs in countries with high burdens of human immunodeficiency virus before patent expiry.^6^ Other strategies, such as compulsory licensing, have had limited success.^7^ Tiered pricing has also been proposed but defining each tier and an appropriate price for each has proved challenging.^8^ The recent approval of high-priced medicines for many conditions has prompted a new round of discussions^9^ and calls for radical changes to the current commercial model for drug development.^10^

We consider that it is time for a global forum to discuss objectivity and equity in access to high-priced drugs. Such a forum should extend beyond the usual networks of payers and authorities on health technology assessment. It needs to define the methods needed to manage the early entry of promising products – i.e. how to evaluate the data that are available for early market entry, determine an appropriate initial price, optimize the collection of data from clinical practice, enable independent trials and manage the exit of products that, in practice, are found to be insufficiently effective. The forum should promote the development of a method for pricing new drugs. Such a method needs to reconcile the need for fair pricing, with the difficulties of obtaining accurate information on research, development and manufacturing costs. The forum should include representatives of patient and consumer groups, so that the right questions are asked, appropriate research priorities are set and outcomes are communicated. 

In managing access to new drugs, simply continuing to react country-by-country and disease-by-disease is not sustainable. We need to be more forward-thinking and take some of the pressure off small purchasers and countries that are currently trying to make equitable decisions in isolation. We need to solve the fundamental problem of how to balance objectivity of appraisal and equity in access to new products; ensuring that medical advances are affordable, working with a viable pharmaceutical industry that responds to public health needs.
